# Supplementary material for: Applications of dry chain technology to maintain high seed viability in tropical climates
Source: PeerJ. 2024 Oct 11;12:e18146. doi: 10.7717/peerj.18146 (PMC11472788; doi:10.7717/peerj.18146)
Supplement: Table S1 — Articles are listed in chronological order. [file peerj-12-18146-s001.docx]

**Supplementary Table 1: Synthetic review of scientific publications on the use of the dry chain approach and zeolite beads for seed drying and conservation. Articles are listed in chronological order.**

“Ratio of beads to seed tested” refers to the ratio between the initial weight of activated zeolite beads and the initial (moist) seed weight. In “Additional observations,” we highlighted the main challenges encountered by the authors in implementing the dry chain and/or the use of zeolite beads. In “Main results,” when referring to “seeds conserved with the dry chain”, we refer to seeds dried with zeolite beads and subsequently stored in hermetic containers.

| **Publication** | **Country/ Location of experiment** | **Study species (Initial MC of seed used in experiment)** | **Ratio of beads to seed tested** | **Drying Protocol** | **Parameters**  **tested** | **Main Results** | **Additional observations** |
| --- | --- | --- | --- | --- | --- | --- | --- |
| Van Asbrouck and Taridno 2009 | NA | Cucumber (13.9%) | 1 | 2 different experiments:  I)Drying with beads for up to 24 hours.  II)Drying with beads compared with sun drying and a conventional drier. | - MC.  -Germination and mean germination time (MGT). | - Seed MC rapidly declined with beads.  - Germination was similar among treatments. | NA |
| Hay et al. 2012 | Philippines, seed laboratory | Rice (>18%) | Different ratios from 0 to 3 | 4 different experiments:  I) Drying with beads, storage without beads.  II) Drying with beads, storage with beads.  III) Drying with beads or silica gel at different ratios.  IV) Drying with beads or silica gel to determine moisture adsorption isotherms. | -MC monitoring and beads water uptake.  -eRH monitoring of beads and seeds.  - Germination. | - Beads showed a higher affinity to water over silica gel particularly at low RH. Beads were able to dry rice seeds to lower moisture content. For drying to moisture contents > 9-10%, silica gel was more efficient than zeolite beads.  - Beads did not work to their full capacity in seed drying.  - Rapid rates of drying achieved using the drying beads, without impairing germination ability and seed quality. | Calculating the quantity of beads to use to reach the target moisture content is not straightforward, a protocol is needed. Risks of over-drying the seed. |
| Kunosoth et al. 2012 | India, seed laboratory | Onion (8.7%), sunflower (9.67%), soybean (12%), mung bean (10.3%) | Authors suggest rates between 0.5 to 1 | Seed stored with beads in hermetic containers for up to 17 months. Compared with undried seeds in porous bags. | -MC.  -Germination.  -Effects of dry chain on pulse beetle infestation in beans. | - Seed MC was reduced in all experiments for seeds stored with beads (<5% onion, 2.4% sunflower, 4% soybean, 3.7% pulse beetle).  - Germination after storage was higher in seeds stored with beads (80% onion, 92% sunflower, 73% soybean, 95% pulse bean), when compared with untreated seeds (41.5%, 45%, 10% and 24% respectively).  - Storage with beads decreased the damage of bruchid infestation . | Drying beads are currently inaccessible for individual farmers. |
| Hay and Timple 2013 | Philippines, seed laboratory | Rice (varying MCs) | Different ratios from 1 to 3 | 3 different experiments:  I) Drying with beads, storage with beads, 3 methods to determine the moisture adsorption isotherm of beads;  II) Fresh or pre-dried seeds dried and stored with beads;  III) Fresh or pre-dried seeds conserved and stored together with desiccants and later separated in different experimental treatments. | -MC monitoring and beads water uptake.  -eRH monitoring of beads and seeds. | -Rehydrating beads followed by passive drying at 15% RH, 15°C, is a reliable method to assess bead capacity to adsorb water from seeds.  - Mixing freshly harvested rice seeds with dry beads at a ratio of 1:1, reduces MC to acceptable levels for long-term genebank storage.  - Pre-drying seeds in dry room conditions (15% RH, 15°C) reduces the quantity of beads required. | At low seed MC the beads become less effective, due to the high proportion of water in the seeds that is held in strong-water-binding sites. |
| Nassari et al. 2014 | India, seed laboratory | Tomato (17%) | Two different ratios: 0.5 and 1 | 5 different conservation treatments:  I Sun drying (control).  II Hermetic container + zeolite beads (1 ratio).  III Hermetic container + silica gel (1 ratio).  IV Hermetic container + zeolite beads (0.5 ratio).  V: Hermetic container + silica gel (0.5 ratio).  Seeds were dried for 96h. | -Seed MC.  -Germination. | - Zeolite beads dried tomato seeds to 4.4% and 7% at 1 and 0.5 beads to seed ratios respectively, after 96 hours.  - Zeolite beads were more effective than silica gel in seed drying (final MC: 7.2% and 8.4% at 1 and 0.5 silica to seed ratios), after 96 hours.  - Zeolite beads and silica gel were more effective than sun drying in seed drying. | NA |
| Ndinya et al. 2017 | Kenya, seed laboratory | Amaranth (13.3%) | 0.41 | - Seeds stored with beads in hermetic containers for 24 months at room temperature. Compared with seeds stored in jute bags. | -Seed eRH.  -Germination. | -Zeolite beads dried amaranth seeds to 6% of MC.  -Seeds stored with beads in hermetic containers maintained initial germination (56%).  - Germination of seeds stored in jute bags declined to 22.6% after 24 months. | NA |
| Bakhtavar et al. 2019 | Pakistan, seed laboratory | Maize (12%) | 0.17, 0.09 | - Seeds dried with beads in hermetic container and then stored in hermetic plastic bags (Super Bags) for 4 months. Compared with seeds with higher MC and/or storage in porous containers. | - MC.  - Germination, Mean Germination Time (MGT), Germination Index (GI).  - Determination of starch, protein and malondialdehyde (MDA) content.  - Grain losses due to lesser grain borer infestation.  - Determination of aflatoxin contamination. | - Zeolite beads decreased MC to 8 and 10% (depending on the ratio), Super Bags maintained low MC in storage.  - Highest germination of seeds conserved with the dry chain after 4 months (92.2%) as well as fastest MGT and highest germination index.  - Highest starch and protein contents, and lowest levels of MDA in seeds conserved with the dry chain (seed drying with beads and conservation in Super Bags).  - Seed stored with the dry chain showed the lowest level of insect infestation.  - No aflatoxin contamination was found in treatments with low moisture contents. | NA |
| Hilli and Vyakaranahal 2019 | India, seed laboratory | Eggplant (8%) | 0.21 | Seeds stored in hermetic containers for 9 months with three different desiccants:  I zeolite drying beads,  II bentonite granules,  III charcoal granules  and seeds stored without desiccants (control). | -MC.  -Germination.  -Seedling vigor index. | -Zeolite beads reduced seed MC to 3.80% lower than other desiccants.  -Seed stored with beads maintained the highest germination and seedling vigor index among the treatments tested after nine months of storage (84.6%). | NA |
| Bakhtavar and Afzal 2020a | Pakistan, seed laboratory | Quinoa (10.2%) | 0.12, 0.01 | Seeds dried with beads in a hermetic container and then stored in hermetic plastic bags (Super Bags) for 18 months. Compared with seeds with higher MC and/or storage in porous containers. | - MC.  - Germination.  - Seed vigor through accelerated ageing.  - Electrical conductivity of seed leachates.  - Seed biochemical attributes (i.e. activity of α-amylase, amounts of reducing and soluble sugars, MDA content). | - Zeolite beads decreased MC to 8% and 10% (depending on the ratio). Super Bags maintained low MC in storage.  - Highest germination for seeds stored with the dry chain.  - Seed vigor higher for the seeds stored with the dry chain.  - Seed stored with the dry chain had lower values of seed leachates.  - Seeds stored in hermetic bags showed maximum α-amylase activity. Maximum quantity of total soluble sugars and minimum quantity of reducing sugars and MDA in seeds stored with the dry chain. | NA |
| Bakhtavar and Afzal 2020b | Pakistan seed laboratory | Wheat  (10%) | 0.05 | Seeds dried with beads in a hermetic container and then stored in hermetic plastic bags (Super Bags) for 4 months. Compared with seeds with higher MC and/or storage in porous containers. | - MC.  - Biochemical analysis of stored seed (i.e. starch, protein and MDA contents).  - Percentage of storage weight loss.  - Determination of aflatoxin contamination. | - Zeolite beads decreased MC to 8%. Super Bags maintained low MC in storage.  - Maximum starch contents, highest protein contents and lowest MDA contents in seed with low MC conserved in Super Bag.  - No insects found and lowest quantitative grain losses for seeds stored in Super Bags.  - No aflatoxins detected for seeds with low MC conserved in Super Bags. | NA |
| Guzzon et al. 2020 | Guatemala, 3 villages in community seed banks and farmers’ houses | Maize (18.4%) | 0.65 | - Seeds dried with beads in hermetic boxes for 8 days.  - Seeds then packaged in hermetic plastic flasks in 3 communities for 6 months, compared with untreated seeds and/or conservation in open storage. | - Moisture content (MC).  - Germination in the lab.  - Seedling emergence and length in sand benches.  - Seed quality (% of infested seeds). | -After eight days of drying using drying beads, the seed MC decreased to 11.5%  - Dry chain maintained very high seed germination >80% (untreated seeds 48%)  - Reduced fungal and insect infestations in treated seeds (<3%). 19% infested in untreated seeds. | Drying beads are currently inaccessible for individual farmers. |
| Kamran et al. 2020 | Pakistan seed laboratory | Cotton (MC varied between cultivars and among picking time from 9.59 to 10.51% | Not stated, based on the online calculator:http://www.dryingbeads.org/?wpdmpro= drying-beads-calculator | - Seeds sun dried for 6h.  - Seeds dried with beads in hermetic plastic box for 1h.  - Seeds then packages in I) cloth bags or hermetically sealed bags at room temperature, II) paper bags at 10°C (cold storage) for 5 months. | - Moisture content (MC).  - Germination in the lab.  - Free fatty acid (FFA) profile.  - Seedling emergence and several other treatments measured in the field (time to start of  blooming, numbers of bolls and sympodial branches, and plant height yield estimate). | - Drying with zeolite beads proved more efficient for lowering MC;  - The decline in germination percentage was least with bead drying and subsequent hermetic storage.  - Minimum quantity of FFA was maintained in seeds dried with zeolite beads and subsequently placed in hermetic storage.  - Plants raised from seed dried with beads and subsequently hermetically stored produced maximum plant height, more bolls and more sympodial branches, and bloomed earlier, producing higher yield. | NA |
| Musebe et al. 2020 | Kenya and Tanzania; Socioeconomic study based on questionnaire of seed producers and traders. | African nightshade; amaranth; common bean; peanut; kale | NA | NA | - Production costs.  - Drying costs (traditional methods and drying beads).  - Storage costs (traditional methods and dry chain).  - Storage losses.  - Selling price.  - Weight of seed. | - The dry chain is currently profitable for large volumes of seeds (e.g. for farmers groups, seed companies and genebanks).  - Crop species for which the dry chain is currently appropriate are the ones for which larger volumes are produced and traded (e.g. common bean). | Drying beads are currently inaccessible for individual farmers. |
| Nivethitha et al. 2020 | India, seed laboratory | Okra (10.5%) | Different ratios: 0.5, 1, 2, 3 | Seed dried with beads and silica gel conserved in hermetic containers. | -Seed MC monitoring.  -Germination.  -Vigor indexes.  - Seedling vigor (length, fresh and dry weight). | -Zeolite beads significantly reduced seeds MC to 6.8, 6.22, 5.22, 4.59 considering the 0.5, 1, 2, 3 beads to seed ratio respectively.  -Zeolite beads dried seeds to lower moisture contents and quicker than silica gel.  -No detrimental effects on seed germination and seedling vigor. | NA |
| Yamalle et al. 2020 | India, seed laboratory | Onion (MC varied among cultivars: 8.46%, 5.25% and 5.75%) | 2 | Seeds dried with beads for 96 h and then stored in poly packs for 12 months. Compared with undried seeds. | - MC.  -Germination.  - Seedling length and dry weight.  - Vigor indexes.  -Electrical conductivity. | -Seed MC was reduced to 3.48%, 3.12% and 2.91%.  - The germination was 80.55% and 51.78% after 12 months for seeds dried with beads and untreated respectively.  - Seedling length and dry weight was higher for seeds ultra-dried with beads.  - Vigor indexes were higher for ultra-dried seeds.  - Ultra-dried seed showed lower electrical conductivity when compared with untreated seeds. | NA |
| Sultana et al. 2021 | India, seed laboratory | Mung  bean (10%) | 0.2 | - Seed stored with beads (or other desiccants) for 9 months in hermetic plastic containers.  - Drying with beads was compared with other desiccants and untreated seeds. | - MC.  - Germination test.  - Seedling length and biomass.  - Ion leakage.  - Detection of storage fungi.  - Effects of drying on infestation after inoculation with bruchid beetles. | - After 48 hours the seed MC decreased from 10% to 6%.  - After 9 months of hermetic storage with beads, 92% of lab germination and 81% of soil emergence.  - Seeds stored with beads showed the highest seedling vigor, the lowest electrical conductivity values after storage, and the lowest frequency of seed borne pathogens.  - Seeds stored with beads were the least affected by introduction of bruchid beetles into the storage containers. | NA |
| Nelwadker et al. 2022 | India, Seed Laboratory | Wheat | 0.37 | - Seed stored with and without beads in polythene bags.  - Seed stored in cloth bags.  Storage period of 12 months. | - MC.  - Insect populations (number of live and dead insects). | -Zeolite beads reduced seed moisture content to 5.65%.  - Storage with beads in polythene bags reduced and delayed insect infestations. |  |
| Singh and Mishra 2022 | India, Seed Laboratory | Groundnut (11.37%) | Different ratios: 1, 0.9, 0.8, 0.7. | Pods of groundnut infested with groundnut bruchid in hermetic containers one week before the treatments: I) mixing with sodium aluminum silicate; II) zeolite drying beads; III) control. Storage period of 6 months. | -MC.  -Seed Germination.  -Bruchids fecundity and adult emergence.  -Weight loss. | - Highest beads ratio was the treatment the reduced the most bruchids fecundity and adult emergence as well as pods weight loss.  -Zeolite beads at the highest ratio reduced MC to 3.37% after 6 months of storage.  - Final germination was highest for seeds conserved with zeolite beads at the highest ratio (73.67%). | NA |
| Trail et al. 2022 | Thailand and USA. The different containers with seeds where stored in porches exposed to ambient conditions | Okra (6%), sorghum ( 9%) and velvet bean ( 12%) | 0.5 | 5 different treatments: I) Seeds stored for 12 months with beads and II) Calcium oxide in a glass jar  III) Seeds stored using electric vacuum sealing machine in polyethylene bags and IV) Under vacuum sealing using a  modified bicycle pump vacuum sealer with glass jars. V) Control seed packaged in paper bags. | -Seeds and desiccants MC.  -Germination. | -Zeolite beads reduced seeds MC to ultradry levels (2% in okra, 2% in sorghum, 4% velvet beans).  - Ultra-drying with beads affected seed germination in okra and velvet bean (final germination of <40% and <20% respectively) but not in sorghum (final germination 75%). | The ratio of beads (conserved with seeds) used for okra and velvet beans was probably too high, detrimentally affecting seed germination. |
| Yahaya et al. 2022 | Malaysia, seed laboratory | Lablab bean (25%) | 1 | Seed dried to target moisture contents of 14%, 12% and 10% by:  I: Sun drying.  II: Shade drying.  III: Oven drying.  IV: Drying beads.  Seed then stored in hermetic aluminum packets for six months at ambient (31°C) and cold (3.5°C) temperatures. | -Seed MC.  -Germination.  -Seedling Vigor Index (SVI).  - Germination Rate Index (GRI).  -Electrical conductivity of leachates.  - Activity of antioxidant enzymes, catalase and peroxidase. | -Seed drying with beads was the fastest among the treatments, seed dried with beads showed the highest germination percentage (71%), speed and vigor when compared to the other 4 treatments.  - Seed germination and vigor decreased at ambient temperature and at increasing moisture contents. Reduced catalase and peroxidase activities at ambient when compared to cold storage. | NA |
