# Supplementary material for: Applications of dry chain technology to maintain high seed viability in tropical climates
Source: PeerJ. 2024 Oct 11;12:e18146. doi: 10.7717/peerj.18146 (PMC11472788; doi:10.7717/peerj.18146)
Supplement: Supplemental Information 3 — Detail methodology of the drying and accelerated ageing experiments performed at CIMMYT (Mexico). [file peerj-12-18146-s003.docx]

*Detailed materials and methods of the experiments carried out in Mexico.*

In order to compare the two drying methodologies, the same quantities of seeds of 21 maize accessions (see Table 2 for the initial weight and MC of the accessions employed in this research) were dried in AF with the use of beads in the drying cabinet as well as in GB in a conventional dry room. Additionally, the seeds of the same accessions and origin dried in AF and GB with the two methods to similar MC (see Table 2 for MC values after drying) were then exposed to accelerated ageing to assess whether the drying with beads had influenced potential seed longevity.

The 21 accessions employed for this experiment were regenerated at the high-altitude CIMMYT research station in Metepec (Toluca, Mexico State) in 2019. These accessions were sown on 14 March 2019, and the harvest occurred between 10 October and 26 November 2019. The harvested cobs were then brought to CIMMYT GB, air dried and shelled, and stored under room conditions in porous containers until the beginning of the experimental phase in January 2020. Just before the start of the comparative drying trials on 20 January 2020, the MC of each accession was tested with three replicates per seed lot using a moisture meter (SL95, Steinlite, Atchison, KS, USA) and the seeds of all accessions were weighed (see Table 2 for initial seed weight and MC data for all accessions) and then divided into two seed lots of the same weight. One seed lot per accession was put in the dry room of GB at 9–15 °C and 10–20 % RH. The other seed lots were brought to AF to be dried with the beads in the drying cabinet. A total of 34.8 kg of seeds were dried with both methods. Drying beads were put in mesh bags, which were placed on the metal grid at the bottom of each section of the drying cabinet; a total of 8 kg of beads were put in Section 0 (see Fig. 4B), 4 kg in the other sections (Sections 1-6, see Fig. 4C) for a total of 32 kg of beads. The seed lots were organized in the upper porous trays in the cabinet (see Fig. 4). The SMC (via the above-mentioned moisture reader) and weight of each seed lot were monitored daily; the RH and temperature inside the cabinet were also monitored every 10 minutes with three dataloggers (DataLogger, Centor Thai) placed in different parts of the cabinet for the four-day duration of the experiment. The mesh bags of drying beads in the cabinet were replaced daily with bags of beads that had been reactivated in the oven of the kitchen of the research station at 200-250°C for 2-3 hours.

At the end of the four days of drying in AF, the seeds were packed in heat-sealed trilaminate aluminum pouches. The average temperature and RH in the drying cabinet at AF were 27.5 ± 2°C and 21.4 ± 5% RH, respectively. The seed lots in the dry room at GB were dried until they reached a MC value within the 95% confidence interval of the MC of the seed lot of the same accessions dried at AF. After reaching the appropriate MC in the dry room, these seed lots were also packed in trilaminate aluminum pouches.

The accelerated ageing experiment was carried out on 12 accessions randomly selected (see Table 2) and started on 31 July 2020 in the Seed Laboratory of the CIMMYT Germplasm Bank (GB). A total of 420 seeds of each of the seeds lots (dried at AF and GB) were placed in open petri dishes randomly interspersed in two 300 × 300 × 130 mm sealed electrical enclosure boxes (Ensto UK Ltd, Southampton, UK) placed in a compact incubator (UN160, Memmert, Germany) at 60% RH and 45°C in the dark. The target 60% RH was controlled by placing the Petri dishes over a LiCl solution prepared following Newton et al. (2009). The RH in the box was monitored with a data logger inside the enclosure box (DataLogger, Centor Thai). When necessary, the bulk solution was adjusted by adding distilled water, stirring and allowing the solution to equilibrate (Hay et al. 2008; Newton et al. 2009).

Germination of each seed lot was tested with duplicates of 30 seeds each at the beginning of the experiment and with seeds retrieved from the boxes after 9, 21, 28, 37, 44 days of ageing. Each replicate was sown in rolled filter paper moistened with distilled water. Filter paper rolls were inserted in transparent plastic trays, and the trays were randomly dispersed in an incubator at a constant temperature of 25°C and a 12 h photoperiod. Distilled water was added to the trays as needed, to avoid desiccation. Germination scoring was performed 1 week after sowing. A seed was considered to be germinated if it had developed into a normal seedling, according to ISTA (2018) criteria (see Guzzon et al. 2021 for the description of the germination protocol employed at the CIMMYT maize collection).

Statistical analyses and data visualization were carried out in R version 4.2.3 and RStudio 2023.06.0. Statistical significance was set at 0.05. Binomial GLMs were used to extract the p50s (the time for viability to decline to 50%) using the probit link function after comparing logit and probit with their AIC (Akaike Information Criterion, AF logit – probit: 6.8, GB logit – probit: 7.5). The p50s for each accession and drying method are presented in Table 2. A binomial GLM with probit link function was also applied to determine the effect of drying method, accession and their interaction on the p50 as longevity correlate. A post-hoc multiple comparison analysis (Tukey) was applied to evaluate the differences between drying method for each of the accessions analyzed.
